# Strategies to improve the therapeutic effect of pluripotent stem cell-derived cardiomyocytes on myocardial infarction

**DOI:** 10.3389/fbioe.2022.973496

**Published:** 2022-08-05

**Authors:** Yang Xiao, Yihuan Chen, Chunlai Shao, Yaning Wang, Shijun Hu, Wei Lei

**Affiliations:** ^1^ Department of Cardiovascular Surgery of the First Affiliated Hospital and Institute for Cardiovascular Science, Collaborative Innovation Center of Hematology, State Key Laboratory of Radiation Medicine and Protection, Suzhou Medical College, Soochow University, Suzhou, China; ^2^ Department of Cardiology, The Second Affiliated Hospital of Soochow University, Suzhou, China

**Keywords:** pluripotent stem cell, cardiomyocyte, myocardial infarction, cell therapy, cardiology

## Abstract

Myocardial infarction (MI) is a common cardiovascular disease caused by permanent loss of cardiomyocytes and the formation of scar tissue due to myocardial ischemia. Mammalian cardiomyocytes lose their ability to proliferate almost completely in adulthood and are unable to repair the damage caused by MI. Therefore, transplantation of exogenous cells into the injured area for treatment becomes a promising strategy. Pluripotent stem cells (PSCs) have the ability to proliferate and differentiate into various cellular populations indefinitely, and pluripotent stem cell-derived cardiomyocytes (PSC-CMs) transplanted into areas of injury can compensate for part of the injuries and are considered to be one of the most promising sources for cell replacement therapy. However, the low transplantation rate and survival rate of currently transplanted PSC-CMs limit their ability to treat MI. This article focuses on the strategies of current research for improving the therapeutic efficacy of PSC-CMs, aiming to provide some inspiration and ideas for subsequent researchers to further enhance the transplantation rate and survival rate of PSC-CMs and ultimately improve cardiac function.

## Introduction

Myocardial infarction (MI), also called heart attack, is the most common form of cardiovascular disease and a leading cause of mortality worldwide (Roth et al., 2020). MI involves the ischemic death of myocardial tissue, usually due to sudden occlusion of the coronary artery caused by plaque rupture and acute thrombosis, resulting in the obstruction of blood flow to a section of the heart. Myocardial cell death triggers an intense inflammatory response and subsequent cardiac fibrosis in the infarction area, ultimately leading to adverse ventricular remodeling (Pfeffer and Braunwald, 1990; Chiong et al., 2011). Notably, patients who have suffered an MI are at increased risk of recurrence or other cardiovascular diseases. Moreover, patients with MI commonly have other diseases (e.g., diabetes) that may further aggravate the symptoms of MI (Schmitt et al., 2021). Despite advances in MI treatment, current therapeutic strategies remain limited to thrombus removal, blood perfusion, and heart transplantation (Lu et al., 2015). However, these methods are either unable to restore necrotic myocardial cells due to the limited regenerative capacity of the adult heart or difficult to apply at a large scale due to the relative lack of heart donors and the high risk of this procedure.

Cell therapy represents a valuable treatment method for MI. It has been shown to promote a certain degree of functional recovery of infarcted hearts in preclinical studies by orchestrating inflammatory reactions, promoting angiogenesis and cell survival, or replacing lost cells with *de novo* cardiomyocytes. Among the cell types currently used for therapeutic purposes are pluripotent stem cells (PSCs), including embryonic stem cells (ESCs) and induced pluripotent stem cells (iPSCs); adult stem cells (e.g., mesenchymal stem cells and epithelial stem cells); and somatic cells (e.g., skeletal myoblasts) (Zhang et al., 2021). While adult stem cells have less ability to differentiate into a limited number of cell types, PSCs have the potential to differentiate into any cell type in the body and thus represent a promising source of functional cells for different therapeutic purposes. Compared with adult stem cells, PSCs showed greater ability to restore cardiac function in a porcine MI model (Ishida et al., 2019).

**FIGURE 1:**
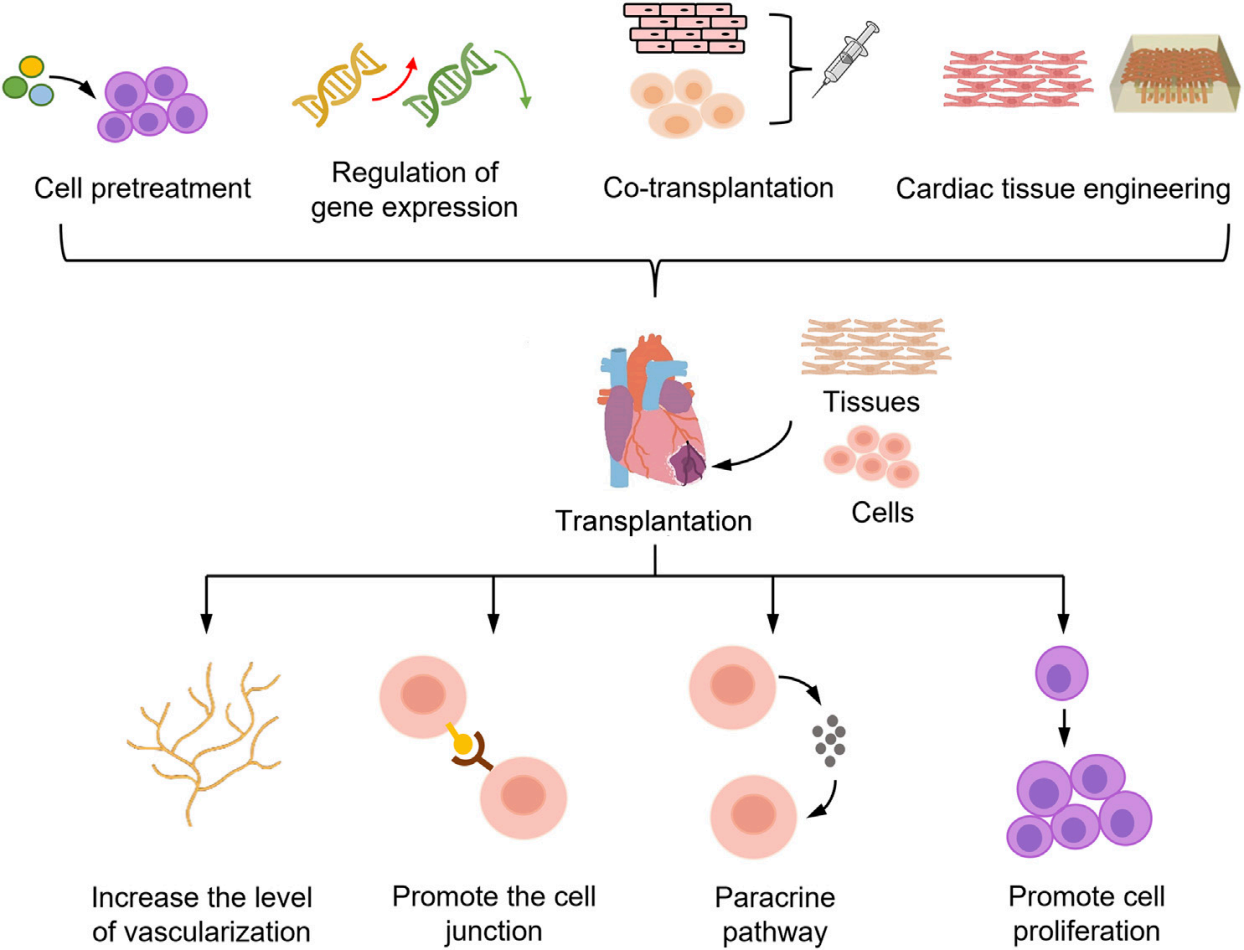
Strategies to enhance the efficacy of PSC-CMs in the treatment of myocardial infarction. PSC-CMs transplanted into the myocardial infarct region die in large numbers during the initial stage of transplantation, limiting the potential of PSC-CMs for cell therapy. To improve the survival rate of PSC-CMs, the main strategies adopted are pretreatment of PSC-CMs, gene expression modulation, co-transplantation with other cells, and cardiac tissue engineering. These strategies mainly aim to increase the number of cells and survival of PSC-CMs after transplantation by improving the vascularization level of grafts, enhancing the connection between donor and host cells, and promoting cell proliferation and paracrine pathways. Ultimately, these strategies can be used to achieve the goal of improving the efficacy of PSC-CMs in the treatment of myocardial infarction.

While ESCs are derived from the inner cell mass of the embryonic blastocyst, iPSCs are similar to ESCs and are generated from somatic cells by the transduction of different reprogramming factors, e.g., c-MYC, Klf4, Oct4, and Sox2. There are potential challenges with the use of iPSCs and ESCs in cell therapy that must be addressed to minimize the drawbacks and enhance the benefits. Transplanted exogenous ESCs often elicit immune rejection, which markedly diminishes the therapeutic effect or causes further damage; immune rejection can be suppressed by HLA matching and knocking down the antigen-encoding gene. In contrast, iPSCs derived from autologous cells do not evoke severe immune rejection by the organism (Zhang et al., 2009). The PSC-derived cardiomyocytes (PSC-CMs) were recently shown to have a significant ameliorative effect on MI, replacing some of the damaged cardiomyocytes and restoring the mechanical contractility of cardiomyocytes after transplantation into the infarcted area (Qiao et al., 2011; Yu et al., 2019; Guan et al., 2020). These data indicate the value of PSC-CMs for the treatment of MI. However, many factors still limit the use of PSC-CMs for MI treatment, including the low transplantation efficiency, short survival time and low survival rate of cardiomyocytes after transplantation, and the possibility of arrhythmia caused by PSC-CM transplantation (Liao et al., 2010). Given the great potential and limitations of PSC-CMs, scientists are developing various strategies to improve the effect of PSC-CMs in the treatment of MI (Figure 1). In this article, we focus on strategies that are currently widely used.

## Cell pretreatment

Faced with the limitations of PSC-CM therapies for MI, one possible strategy is to pretreat PSC-CMs before transplantation. Many drugs have been shown to be efficacious in MI, which has led investigators to wonder whether the pretreatment of PSC-CMs with these drugs could improve their efficacy in the treatment of MI. Additionally, pretreatment with non-drug factors, such as hypoxia and starvation, has also been shown to somewhat alleviate the limitations of PSC-CM therapy.

If medications are used for treatment, the first goal is to select the right drug. For example, drugs that increase cell proliferation and promote cell survival in the heart (such as the FGF family and CHIR99021) may be promising. The fibroblast growth factor (FGF) family, also known as the heparin affinity growth factor family, is a class of peptide-like molecules that bind to specific cell membrane receptors. Fibroblast growth factor 1 (FGF1) is involved in several physiological processes, such as development, morphogenesis and proliferation, and FGF1 treatment for acute myocardial injury was found to increase cardiomyocyte mitosis, reduce scar area, and improve cardiac function (Beenken and Mohammadi, 2009). FGF21 also affects a variety of physiological processes, including regulating glucose and lipids and reducing large vessel atherosclerotic plaque formation, and has significant protective effects against MI, myocardial ischemia–reperfusion injury and diabetic cardiomyopathy (Tanajak et al., 2015). There is evidence that FGF21 improves inflammation and myocardial fibrosis in MI via the early growth response protein 1 (EGR1) (Li et al., 2021a). CHIR99021, a potent and highly selective inhibitor of glycogen synthase kinase 3 (GSK3), enhances cell cycle activity by inhibiting GSK3. CHIR99021 can promote PSC-CM proliferation and maintain their immature state; upon removal of CHIR99021, the cells can return to a normal myocardial state. One *in vivo* study in a mouse model found that transplantation of human iPSC-CMs into myocardial infarct areas after combination pretreatment with CHIR99021 and FGF1 increased cell cycle activity by approximately 4–6-fold and the transplantation rate by approximately 4-fold. The effect of CHIR99021 or FGF1 pretreatment alone was weaker than that of cotreatment but significantly stronger than that of the control (Fan et al., 2020).

There are also drugs that promote vascularization and graft–host cell integration, such as thymosin β4 and Y-27632. Thymosin β4 (Tb4) was found to promote cardiomyocyte survival and proliferation and to enhance angiogenesis. Tan et al. demonstrated *in vitro* that Tb4 could increase AKT activity and BcL-XL expression to protect cultured hiPSC-CMs from hypoxic injury. In a porcine MI model, the combined effect of human iPSC-CMs and Tb4 improved the engraftment rate, vascularization and cardiac function, and no tumor tissue was found in this immunodeficient porcine model (Tan et al., 2021). The enhancement of cellular junctions between transplanted cells and host cells contributes to the survival of transplanted cells. Zhao et al. found that pretreatment of iPSC-CMs with a Rho kinase (ROCK) inhibitor, Y-27632, significantly improved the transplantation rate and the therapeutic effect on MI. ROCK regulates cytoskeletal changes associated with cell adhesion, suggesting that cell–cell junctions play an important role in the therapeutic effect of transplanted PSC-CMs in the MI region (Zhao et al., 2019). Y-27632 is commonly used to promote cell survival and wall attachment during cell passaging.

Faced with the problems of arrhythmia and tachycardia caused by the transplantation of PSC-CMs, one feasible option is to apply drugs to regulate the electrical phenotype of PSC-CMs. Liu et al. found that canonical transient receptor potential isoform 7 (TRPC7) channels play an important role in regulating the automaticity of ESC-CMs. TRPC7 is a receptor-operated Ca^2+^ channel that can be activated by binding to DAG and mediates Ca^2+^ influx, indicating that TRPC7 is an important potential drug target (Liu et al., 2021). Similarly, it has been suggested that TRPC3 has a similar role to TRPC7 in regulating the automaticity of ESC-CMs (Qi et al., 2016), and the identification of drugs targeting these targets could address the problem of rapid arrhythmias after transplantation and improve the therapeutic efficacy of ESC-CMs.

The basic principle of nondrug pretreatment is that unfavorable environments, such as hypoxia and starvation, activate cellular resilience and force PSC-CMs to enhance the utilization of oxygen and nutrients. Yang et al. treated PSC-CMs with Earle’s balanced salt solution (EBSS) to simulate starvation *in vivo* and found that EBSS-induced starvation significantly promoted the structural, metabolic and electrophysiological maturation of PSC-CMs. Moreover, this treatment increased the mitochondrial content of ESC-CMs and enhanced their oxidative phosphorylation capacity (Yang et al., 2021). ESCs are derived from the inner cell mass of the embryonic blastocyst stage, and the oxygen content in the embryo is typically 3–5%, which is lower than that in the external environment. Hypoxic conditions play a very important role in the maintenance of stem cells, and hypoxia-inducible factors (HIFs) regulate the pluripotency of PSCs. Among HIFs, HIF-2α is an upstream regulator of Oct4, and knockdown of HIF-2α leads to the downregulation of Oct4, Nanog and Sox2 (Szablowska-Gadomska et al., 2011). Hu et al. injected mouse mesenchymal stem cells (MSCs) into myocardial infarct areas after pretreatment under hypoxic conditions (0.5% oxygen) for 24 h. The results showed that hypoxic pretreatment increased the expression of proangiogenic and prosurvival factors, such as angiopoietin-1 (Ang-1), vascular endothelial growth factor (VEGF) and erythropoietin. Thus, hypoxic pretreatment enhanced the ability of MSCs to repair the infarcted myocardium, reduced the apoptosis of transplanted cells, and increased angiogenesis (Hu et al., 2008). Therefore, unfavorable environmental factors, such as hypoxia and starvation, can somewhat improve cell viability, and such nondrug pretreatments are feasible options for improving the survival rate of PSC-CMs after transplantation.

## Regulation of gene expression

Adult mammalian cardiomyocytes have almost no proliferative capacity; thus, it is difficult to replenish new cardiomyocytes after injury, an important reason for the dramatic impairment of cardiac function after MI. In contrast, during mammalian fetal development, cardiomyocytes have the ability to proliferate, but soon after birth, their proliferative capacity begins to decline until it is lost. This loss of proliferative ability mainly stems from the downregulation of cell cycle maintenance factors as the heart matures, which causes cardiomyocytes to enter G0 of the cell cycle and thus fail to provide new cells at the site of injury after MI. Therefore, researchers have explored ways to increase the proliferative capacity of PSC-CMs from the perspective of gene expression regulation. Overexpressing cell cycle maintenance factors in PSC-CMs may prolong cell cycle progression, allowing more cardiomyocytes to remain in the proliferative phase and increasing the retention of transplanted cells in the infarct zone. Cell cycle protein D (cyclin D) regulates the cell cycle at the G1/S transition, wherein it binds to Cyclin Dependent Kinase 1 (CDK1) and activates CDK1 activity through a series of phosphorylation and dephosphorylation events to move cells from G1 to the replicative S phase. Zhu et al. showed that the overexpression of cyclin D2 in human iPSC-CMs increased graft size and improved myocardial recovery in a mouse model of MI by increasing the proliferation of transplanted cells, which played a significant role in myocardial remyelination and ventricular function recovery (Zhu et al., 2018). Zhao et al. also reported the same ability of cyclin D2 to improve cardiac function in a porcine model of MI (Zhao et al., 2021).

An increased number of PSC-CMs lingering in the infarct zone and increased cell cycle activity can also be achieved by reducing cell death. Many genes are closely related to cell aging and apoptosis, and regulating the expression of these genes can slow cell aging and inhibit apoptosis. Engel et al. showed that p38 activity is one important component of cell cycle regulation in the mammalian myocardium. p38 activity is negatively correlated with cardiac growth, and p38 overexpression inhibits myocardial proliferation (Engel et al., 2005). p38 belongs to the superfamily of mitogen-activated protein kinases (MAPKs), which mediate multiple signaling pathways that regulate cardiomyocyte death (Engel et al., 2006). Fiedler et al. inhibited MAP4K4 activity in hiPSC-CMs by treatment with specific pharmacological agents and confirmed that MAP4K4 inhibition could effectively protect cardiomyocytes from lethal damage (Fiedler et al., 2019).

In addition to increasing the number of PSC-CMs in the MI region by promoting cell proliferation and reducing apoptosis, enhancing the integration between transplanted PSC-CMs and host cells can also improve the therapeutic effect on MI. By expressing certain exogenous genes in PSC-CMs, graft vascularization and cellular junctions between donor and host cells can be improved to achieve functional integration. Tao et al. transfected Ang-1 into human iPSC-CMs and then implanted these genetically altered cells into the anterior wall of the left ventricle after MI in rats. The results showed that Ang-1 improved the survival rate of iPSC-CMs after transplantation, induced mitosis and small artery formation in both host and donor cells, and improved the cell engraftment rate (Tao et al., 2021). Lou et al. found that N-cadherin (CDH2) mediates the adhesion between cardiomyocytes. Therefore, they overexpressed the CDH2 gene in iPSC-CMs and found that CDH2 overexpression improved the therapeutic effect of iPSC-CMs in a mouse model of MI (Lou et al., 2021).

In addition, scientists need to find ways to improve the therapeutic efficacy of PSC-CMs by mitigating problems such as heart rate irregularities that may result from the immature electrical phenotype. The ion channel Kir2.1 is encoded by the KCNJ2 gene and forms inward rectifier potassium current (I_K1_) channels in a variety of cell types, including cardiomyocytes; therefore, Liao et al. overexpressed the ion channel Kir2.1 in ESC-CMs. I_K1_ can hyperpolarize the resting membrane potential, and I_K1_ expression seems to be a marker of ESC-CM maturation. Although ESC-CMs improved cardiac function after MI with or without the overexpression of Kir2.1, the risk of ventricular tachyarrhythmias and sudden death was significantly reduced by Kir2.1 overexpression (Liao et al., 2013).

## Cotransplantation

The heart is not entirely composed of cardiomyocytes but also includes a variety of tissues and cells, such as blood vessels, connective tissue, smooth muscle cells (SMCs), fibroblasts, and endothelial cells (ECs). Therefore, one idea for improving the therapeutic effect after MI is to transplant PSC-CMs into the infarcted area together with other cells or tissues that could ensure a high survival rate of the transplanted PSC-CMs through paracrine signaling and provide a suitable microenvironment for PSC-CMs. This approach has been shown to significantly improve the therapeutic efficacy of PSC-CMs in MI. Sekine et al. cocultured ECs with cardiomyocytes from newborn mice, and these ECs formed a network structure in cardiomyocyte sheets that significantly increased vascular structure and cytokine secretion; in addition, engineered myocardial tissue consisting of three layers of cardiomyocyte sheets was transplanted into a MI model and significantly improved cardiac function (Sekine et al., 2008). This finding indicates that coculture with ECs increases vascularization in grafts, suggesting that coculturing ECs with PSC-CMs may have similar effects.

Cotransplantation of PSC-CMs with other cells or tissues improves cardiac function through two main ways: promoting vascularization and increasing the secretion of paracrine factors. Vascular regeneration of the graft in the infarct area can provide oxygen and nutrients to the transplanted cells and improve the survival rate of transplanted PSC-CMs. Moreover, the graft provides a favorable microenvironment that promotes cardiomyocyte proliferation, survival, and vascularization by prolonging the secretion of paracrine factors. Park et al. used a patch of human MSCs in conjunction with iPSC-CMs to promote myocardial repair (Park et al., 2019). MSCs secrete a variety of paracrine factors, such as VEGF, fibroblast growth factor 2 (FGF2) and hepatocyte growth factor (HGF), which promote angiogenesis, neovascularization and cell survival. The human MSCs also secrete potent antifibrotic factors (e.g., matrix metalloproteinases) that can inhibit the proliferation of cardiac fibroblasts and thus attenuate fibrosis (Uemura et al., 2006).

Paracrine effects are important for the survival of PSC-CMs after transplantation; these cells exert paracrine effects mainly through exosomes, which contain a variety of bioactive proteins, RNAs and microRNAs that act as cytokines to regulate numerous intra- and intercellular processes. It was reported that exosomes secreted by iPSC-CMs can restore the cell loss caused by MI in pigs, and *in vitro* experiments showed that exosomes protect iPSC-CMs and promote vascularization by inhibiting apoptosis, maintaining intracellular calcium homeostasis, and increasing adenosine 5′-triphosphate synthesis (Gao et al., 2020). Therefore, the transplantation of exosomes together with PSC-CMs into the heart is another feasible approach to mitigate myocardial injury after heart attack.

Transplanted cardiomyocytes die in large numbers in the early posttransplant period due to ischemia, suggesting that enhanced local vascularization and blood perfusion may promote graft cell survival. The importance of vascularization for cardiomyocyte survival *in vivo* has been demonstrated in several publications (Stevens et al., 2009). Sun et al. injected microvessels isolated from adipose tissue with human iPSC-CMs into a rat model and found that these microvascular grafts increased vascularization and vascular density in the heart and could be maintained in the heart for a long time. Grafting microvessels with iPSC-CMs improved the survival rate of iPSC-CMs by approximately 6-fold, increased the graft area by approximately 5.3-fold, promoted the maturation of iPSC-CMs *in vivo* and restored a portion of the lost ventricular function after MI (Sun et al., 2020).

When transplanting different cell types into the infarcted area, it is important to consider that different cell proportions may exert different therapeutic effects. In the normal heart, the proportion of cardiomyocytes is the largest and can even reach 80% of the total volume of the heart. Thus, when co-transplanting, the cells should be mixed in the appropriate ratio. Giacomelli et al. constructed a scaffold-free structure of microtissues (MTs) using PSC-derived cardiovascular cells in a ratio of 15% endothelial cells, 70% cardiomyocytes, and 15% fibroblasts, showing that PSC-CMs in MTs have improved sarcomere structures with T-tubules, increased contractility, and more mature electrophysiological phenotypes than PSC-CMs alone (Giacomelli et al., 2020). The main cell types in the heart have significant heterogeneity, with different proportions of cells in different regions (Litvinukova et al., 2020). There are also studies that point to differences in healthy hearts between men and women. The proportion of cardiomyocytes in the heart is higher in women than in men, and factors such as the region of infarction and the gender of the patient should be taken into account in the future clinical translation of cell therapy.

## Cardiac tissue engineering

Myocardial regeneration strategies have been hampered by the lack of cardiac cell sources and the marked loss of donor cells after transplantation. PSC-CMs are a potential adequate cell source for cell replacement therapy but do not solve the problem of rapid donor cell loss. Cardiac tissue engineering is an interdisciplinary strategy in which bionic scaffolds and seed cells are used to generate tissue replacements aimed at restoring the structure, morphology, and function of damaged cardiac tissue (Cho et al., 2021). Commonly used bionic scaffolds include cardiac patches, hydrogels, and nano/microparticles that can serve as carriers of drugs, proteins, and biologically active substances to provide a suitable extracellular environment for stem cells and improve cell survival after transplantation (Nelson et al., 2011; Li et al., 2012). Cardiomyocyte patch technology allows for the delivery of large numbers of cells to the damaged myocardium without the loss of transplanted cells or damage to the host myocardium. Traditional methods of cardiomyocyte patch fabrication include suspending cells within a scaffold composed of biocompatible materials or growing two-dimensional sheets that are stacked to form multilayer structures. Recently, advanced techniques such as micropatterning and three-dimensional (3D) bioprinting have allowed for more precise and standardized preparation of cardiomyocyte patches (Wang et al., 2021). The use of autologous myogenic cell patches was previously shown to improve cardiac function (Memon et al., 2005). Kawamura et al. prepared human iPSC-CMs patches and validated their therapeutic activity using a porcine ischemic cardiomyopathy model; these patches restored normal cardiac function by enhancing cardiomyocyte and electromechanical integration (Kawamura et al., 2012). Gao et al. induced the differentiation of iPSCs into smooth muscle cells, cardiomyocytes and endothelial cells and used these three cell types to form cardiomyocyte patches. Such patches had a better therapeutic effect than iPSC-CMs transplanted alone (Gao et al., 2018).

The developmental maturation of cardiomyocytes requires interactions among various regulatory mechanisms within the cell, but the extracellular matrix also plays an important role (Miao et al., 2020). Samura et al. found that components of the extracellular matrix, especially laminin-221, may play important roles in the survival of human iPSC-CMs after transplantation. Laminin-221 enhanced the mechanical and metabolic functions of iPSC-CMs and improved the therapeutic effects of 3D engineered cardiac tissue (ECT) in a rat ischemic cardiomyopathy model (Samura et al., 2020). Yokoyama et al. encapsulated human iPSC-CM-derived 3D cardiac tissue with the extracellular matrix protein fibronectin and implanted this tissue at the site of MI in rats. The results showed that these iPSC-CMs had better tolerance to hypoxia, improved cardiac injury associated with MI, and had the potential to improve the survival and treatment outcomes of patients with ischemic heart disease (Yokoyama et al., 2021).

The development of cardiomyocytes requires an extracellular matrix with appropriate topological properties, and several studies have shown that a stiffer matrix can better recapitulate the *in vivo* myocardial environment and promote the maturation of PSC-CMs, with better myocardial myofibrillar alignment and more ordered electrical properties (Bhana et al., 2010). After transplanting ECT with iPSC-CMs encapsulated in different matrices to MI sites, many investigators found that these matrices promoted the coupling of iPSC-CMs to host cells. Li et al. developed an injectable hydrogel matrix with hyaluronic acid (HA) as the skeleton that was tailored by introducing gold nanoparticles (AuNPs) and RGD adhesion peptides; these modifications enhanced the biophysical properties of the HA matrix gel and provided adhesion sites for cells. The iPSC-CMs were eventually encapsulated into the prepared hydrogel matrix and injected into the mouse heart post infarction. The transplanted iPSC-CMs formed more mature gap junctions in the ischemic myocardial tissue and improved left ventricular electrical conduction in this murine infarction model (Li et al., 2021b).

In addition, a large number of investigators have constructed ECT using ESC-CMs, which also promote remyelination and revascularization of the infarcted myocardial region and achieve improved cardiac function. Caspi et al. demonstrated that ECT constructed from ESC-derived cardiomyocytes, endothelial cells and embryonic fibroblasts has cardiac-specific ultrastructural, molecular and functional properties and is highly vascularized (Caspi et al., 2007). Lesman et al. inoculated ESC-CMs together with ECs (human biliary vein endothelial cells) and embryonic fibroblasts onto biodegradable porous scaffolds. The resulting ECTs were transplanted into rat hearts and formed cardiac tissue grafts, and studies showed that the transplanted tissues successfully and functionally integrated with the host coronary arteries with a higher level of vascularization than ESC-CMs transplanted alone (Lesman et al., 2010).

The alignment of the cells used for cardiac tissue engineering affects the effect of repair on the heart. Jamaiyar et al. inoculated co-cultured ECs and SMCs in an alignment similar to that of blood vessels on a micro-scaffold made from poly (lactic-co-glycolic acid), and then transplanted induced vascular progenitor cells (iVPC) together with 3D cell/polymer micro-beams formed by micro-scaffolds into a mouse model of myocardial infarction, and the results showed that iVPC on microbeam scaffolds had higher survival rates and the ability to treat infarction (Jamaiyar et al., 2017). Similarly, PSC-CMs can be inoculated onto the scaffold according to the arrangement in the heart. The similar cellular alignment makes it easier for PSC-CMs to bind to the host myocardium and also have more similar beating frequency and amplitude of the host myocardium, thereby reducing the risk of post-transplant arrhythmias. On the other hand, this specific structure also provides some mechanical support for the transplanted cells, allowing better binding of the transplanted PSC-CMs to the host and improving the success rate of transplantation.

## Conclusion and perspectives

PSC-CMs can provide a suitable cell source for cell replacement therapy. Transplanting PSC-CMs into the heart can restore a portion of the cell loss and scar tissue caused by MI, thus restoring normal physiological function of the heart (Sayed et al., 2016). A major obstacle to cell-based cardiac therapy is the extremely low retention and colonization rate on the host myocardium, which is particularly important because the extent of cell-based cardiac repair is largely dependent on the number of cells that survive and colonize the heart (Ban et al., 2014). It is widely recognized that the retention rate of cardiomyocytes delivered to the heart is incredibly low (Zhang et al., 2001). The survival rate after injection is only 50%, dropping to 10% in as little as 1 week (van Laake et al., 2008). The maximum survival time of human PSC-CMs after direct injection into the mouse myocardium has been reported to be no more than 12 weeks. Along with the number of cells transplanted, the maturity of the cells is an important aspect that affects the effectiveness of the treatment. Many studies have also used various methods to improve the maturity of transplanted cardiomyocytes, and it has been shown that immature cardiomyocytes have a risk of inducing arrhythmias.

Therefore, to address these problems, scientists have used strategies such as PSC-CM pretreatment, gene expression modulation, cotransplantation with other cells and cardiac tissue engineering to mitigate the limitations of PSC-CM therapies and increase their efficacy in the treatment of MI. Other investigators have developed combination strategies that can achieve better results. Kawamura et al. found that combining cell sheets with the Omental Flap Technique enhanced the therapeutic effect of human iPSC-CMs in a porcine ischemic cardiomyopathy model (Kawamura et al., 2017). Alternatively, a combination of the above strategies may better exploit the potential of PSC-CMs compared to their use alone; such combinations as treating PSC-CMs with bioactive factors or regulating gene expression before cotransplantation with other cells or constructing engineered heart tissue await validation in future studies.

With regard to the clinical translation of autologous stem cell therapy in acute myocardial infarction, two cell types have now completed phase III clinical trials: skeletal myogenic cells and bone marrow mononuclear cells. Large-scale randomized clinical studies have also demonstrated the clinical feasibility and safety of transcoronary infusion of autologous bone marrow-derived mononuclear cells and circulating progenitor cells in patients with acute MI, showing a 9.3% increase in left ventricular ejection fraction and a reduction in myocardial infarct size at 12 months follow-up (Leistner et al., 2011). Ideally, the best cells for regenerating the heart should be precursors of all cardiac lineages, as iPSCs combine the pluripotency of ESCs with the advantages of autologous use, making them a promising source of cells. ESCs are likewise an alternative stem cell, but issues with ESC sources limit their ability to be used clinically. In clinical applications, fibroblasts from patient blood or urine are collected and differentiated to form autologous iPSCs, which are then induced to differentiate into cardiomyocytes. After differentiation to obtain cardiomyocytes, various strategies of treatment, such as treatment with various drugs, co-culture with other cells and preparation of engineered heart tissues, are adopted to improve the maturation and therapeutic potential of iPSC-CMs, and then the treated cardiomyocytes or engineered heart tissues are transplanted to the infarcted area to restore the number of cardiomyocytes in the injured area.

Current research on PSC-CM replacement therapy is limited to animal and *in vitro* experiments; most studies have used mouse, rat, and porcine models of MI, with few performed in primates. The clinical relevance of animal models is limited by significant physiological differences in size, electrical conduction, and coronary artery formation and distribution between the animal and human heart. Therefore, rigorous clinical trials and the establishment of standard operating procedures are needed to address the safety issues associated with PSC-CM transplantation, such as whether this procedure will cause new heart diseases or lead to tumorigenesis. In addition, the ethical issues associated with the transplantation of PSC-CMs are a major impediment to clinical use. Meanwhile, a standardized and scaled-up culture protocol for PSC-CM treatment in clinical settings is still lacking, and further collaborative research by researchers and medical professionals is needed. Future issues to be addressed in clinical trials may also include determining the appropriate timing of transplantation and the number of cells transplanted. The appropriate number of cells to be transplanted must be determined to ensure efficacy without causing cardiac arrhythmias due to excessive cell numbers. In the future, these strategies can be combined with conventional pharmacological and surgical treatments (e.g., antithrombotic drugs, revascularization, and installation of left ventricular assist devices), which may further improve the therapeutic efficacy of PSC-CMs.

When performing cell transplantation, some undifferentiated and partially differentiated PSCs might be also transplanted together with PSC-CMs into the infarcted region. Since undifferentiated PSCs exhibit intrinsic tumorigenic properties that allow them to form teratomas, cell therapy products containing residual undifferentiated PSCs may lead to tumor formation after transplantation. It has been shown that transplantation of different iPSC lines shows significant differences in tumor incidence, latency of formation, and volume. Cho et al. developed a PSC-specific fluorescent probe, CDy1, which generates reactive oxygen species (ROS) upon visible light irradiation that induces selective PSCs death. Importantly, CDy1 and light irradiation did not have a negative effect on differentiated endothelial cells. Photodynamic treatment of PSCs with CDy1 and visible light irradiation confirmed the inhibition of teratoma formation in mice (Cho et al., 2016). Studies on the inhibition of teratoma formation by PSCs are being further explored, but this risk still cannot be completely avoided. When using PSCs for treatment in the future, it may be advisable to try to ensure complete differentiation of PSCs into cardiomyocytes before using them for treatment.

Although PSC-CMs still predominantly act in a paracrine manner due to low transplantation efficiency and many issues remain to be resolved to achieve clinical translation, the great potential for the treatment of MI encourages researchers to continue exploring and developing better protocols. We believe that with the optimization of PSC-CM pretreatment and transplantation techniques, the goal of PSC-CM transplantation to repair the necrotic myocardium will eventually be achieved.
